# National Economic Burden Associated with Management of Periodontitis in Malaysia

**DOI:** 10.1155/2016/1891074

**Published:** 2016-03-16

**Authors:** Tuti Ningseh Mohd Dom, Rasidah Ayob, Khairiyah Abd Muttalib, Syed Mohamed Aljunid

**Affiliations:** ^1^Department of Dental Public Health, Faculty of Dentistry, Universiti Kebangsaan Malaysia, Jalan Raja Muda Abdul Aziz, 50300 Kuala Lumpur, Malaysia; ^2^Oral Health Division, Ministry of Health Malaysia, 62584 Putra Jaya, Malaysia; ^3^Faculty of Dentistry, SEGi University, No. 9, Jalan Teknologi, Taman Sains Selangor, Kota Damansara, PJU 5, 47810 Petaling Jaya, Selangor, Malaysia; ^4^International Centre for Casemix and Clinical Coding, Faculty of Medicine, Universiti Kebangsaan Malaysia, Jalan Yaacob Latiff, 56000 Cheras, Kuala Lumpur, Malaysia; ^5^Department of Health Policy and Management, Faculty of Public Health, Kuwait University, P.O. Box 24923, 13110 Safat, Kuwait

## Abstract

*Objectives*. The aim of this study is to estimate the economic burden associated with the management of periodontitis in Malaysia from the societal perspective.* Methods*. We estimated the economic burden of periodontitis by combining the disease prevalence with its treatment costs. We estimated treatment costs (with 2012 value of Malaysian Ringgit) using the cost-of-illness approach and included both direct and indirect costs. We used the National Oral Health Survey for Adults (2010) data to estimate the prevalence of periodontitis and 2010 national census data to estimate the adult population at risk for periodontitis.* Results*. The economic burden of managing all cases of periodontitis at the national level from the societal perspective was approximately MYR 32.5 billion, accounting for 3.83% of the 2012 Gross Domestic Product of the country. It would cost the nation MYR 18.3 billion to treat patients with moderate periodontitis and MYR 13.7 billion to treat patients with severe periodontitis.* Conclusion*. The economic burden of periodontitis in Malaysia is substantial and comparable with that of other chronic diseases in the country. This is attributable to its high prevalence and high cost of treatment. Judicious application of promotive, preventive, and curative approaches to periodontitis management is decidedly warranted.

## 1. Introduction

Studies of economic burden of diseases are useful for planning health budgets for a nation. Information from these studies can provide baseline comparisons for new strategies, priority setting, and projection of future cost of running particular health programmes. Perhaps more importantly, an estimate of a disease economic burden may be used to convince health administrators and policy-makers of the magnitude of a particular disease and encourage greater engagement in its prevention or early detection. For example, many chronic diseases such as diabetes and cardiovascular diseases exert considerable economic impacts on health care systems, societies, and the individual patients through the need for continued care and loss of productivity [1–4]. These diseases have common lifestyle-associated risk factors such as unhealthy diet and physical inactivity, which are preventable hence bring about reduction of avoidable costs associated with treatment. Related, some of these diseases have established associations with periodontal diseases [5].

Periodontal diseases are another example of a chronic condition which is largely preventable. Yet its prevalence has been reported to be as high as 90%, while in its severe form, periodontitis has been reported to affect up to 15% of the global population [6]. Of late, there are reports of increasing trends of severe periodontitis in some parts of the world [7, 8]. The recent report on the Global Burden of Disease Study indicated that severe periodontitis affects up to 11% of the global adult population (equivalent to 743 million people) and is the sixth most prevalent disease in the world; this is ranked higher than chronic diseases such as cardiovascular diseases [9]. In this report, periodontitis was defined as either one of the following: a Community Periodontal Index score of 4, a clinical attachment loss of more than 6 mm, or a gingival pocket depth of more than 5 mm. In the same study, severe periodontitis has been cited to have a mean disability-adjusted life year (DALY) which was ranked at number 77. Disability was defined as “bad breath, a bad taste in the mouth, and gums that bleed a little from time to time, but this does not interfere with daily activities.”

Periodontitis, like any other oral conditions, on its own does not cause death. However, its known links with known noncommunicable diseases such as diabetes and cardiovascular diseases [10, 11] increases its role in contributing to the disease burden of these systemic conditions which may bring about fatality. A recent review of periodontitis patients dental records in Malaysia indicated that at least a quarter of these patients suffer from diabetes mellitus and hypertension [12]. In the light of all this current evidence, the dire need for a multidisciplinary approach for these related diseases must be emphasised so that patients are given optimal, timely, and holistic care. Regrettably, in the scarcity of studies on economic impacts of periodontal diseases, the urgency for such care is yet to be realised and acted upon. An estimate of economic burden of managing periodontitis will assist in allocation of resources, provide an economic framework for evaluation of related healthcare programmes, and justify resources for prevention and early detection of the disease. While there are studies estimating costs of periodontal care, the focus had been on cost of specific periodontal treatment modalities but not the cost of managing the whole spectrum of the disease itself [13–15]. The aim of this study is to estimate the economic burden associated with the management of periodontitis in Malaysia from the societal perspective.

## 2. Methods

### 2.1. Ethics

We obtained permission to conduct the study from the respective Institutional Review Boards of the Ministry of Health, Malaysia, and Universiti Kebangsaan Malaysia. Five selected specialist periodontal clinics within the purview of the Ministry of Health were randomly selected according to their locations.

### 2.2. Framework to Determine the Economic Burden

**Figure 1:**
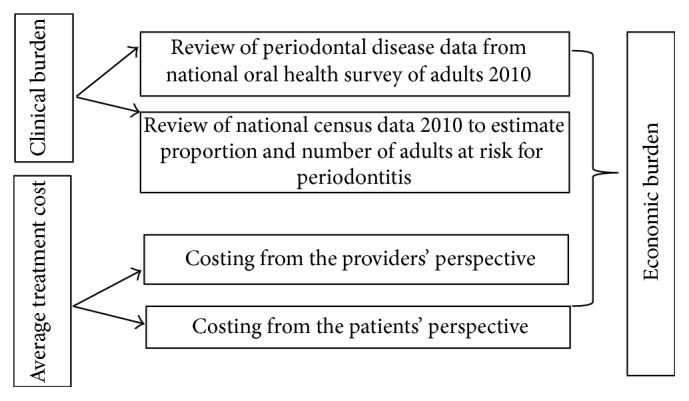


In this study, we estimated the economic burden of periodontitis by combining the clinical burden (prevalence) of the disease with its treatment costs. We set up the data frameset from the following sources of data (Figure 1): (1) the National Oral Health Survey for Adults, 2010 [16], to estimate the prevalence of periodontitis, (2) the National Population Census Data [17], to estimate the number of adults population at risk for periodontitis, and (3) the cost of managing per patient with periodontitis at the public sector specialist periodontal clinics from the perspectives of the health care providers and patients [18].

### 2.3. The Prevalence of Periodontitis

We extracted information on periodontitis prevalence from Malaysia's National Oral Health Survey for Adults [16]. In Malaysia, the Ministry of Health conducts oral health surveys of adults aged 15 years and above once in every ten years. These surveys utilised two-stage sampling technique; each time the probability sampling was based on national census data of enumeration blocks (EB) from the Department of Statistics, Malaysia. Selected government dentists with postgraduate qualifications in dental public health performed the clinical examination after undergoing comprehensive standardisation and calibration sessions. The most recent survey took place in the year 2010. Periodontal assessments used the Community Periodontal Index (CPI): Score 0 = healthy periodontal conditions, Score 1 = gingival bleeding, Score 2 = gingival bleeding and calculus, Score 3 = shallow periodontal pockets (4-5 mm), Score 4 = deep periodontal pockets (≥6 mm), Score 9 = excluded, and Score *X* = not recorded or not visible. We estimate periodontitis prevalence based on survey participants who scored 3 and 4. From the survey, almost half (48.5%) of the participants had periodontitis: 30.3% had moderate periodontitis, while 18.2% had severe periodontitis [19].

The sample size of this oral health survey was 9,065 Malaysians aged 15 years and above which was weighted to represent 88.7% of all Malaysians in the same age group. Majority were females (55.7%), were from the urban (57.9%), were of Malay ethnicity (58.6%), and completed college education (48.9%). Younger adults aged 15–34 years made up 41.5% of the study population.

### 2.4. The Number of Adult Population at Risk for Periodontitis

We used national census data for year 2010 to identify the number of adults aged 15 years and above to estimate the population at risk for periodontitis. In the year 2010, the total population of Malaysia was 28.3 million. The proportion of adults aged 15 and above was 84%, which is equivalent to 23.8 million people.

### 2.5. Cost of Managing a Patient with Periodontitis

We retrieved patient data for the cost estimation and treatment outcomes based on treatment conducted for the 165 periodontitis patients recruited at the participating specialist clinics. The treatment was performed for one year upon commencement. To estimate the cost of managing a patient with periodontitis, we combined two costing methods, namely, the step-down and activity-based costing (ABC) methods. We acquired cost data, administrative and financial records for year 2012 from participating specialist periodontal clinics, national annual reports, and observation of 60 patients undergoing various periodontal treatments to ascertain personnel, time, equipment, and materials consumed. We measured costs using 2012 Malaysian Ringgit (MYR) values. We conducted the cost analysis from the societal perspective: the economic viewpoint of the provider, Ministry of Health, Malaysia, and the patients. Details of the costing methodology are explained in a previous publication [18].

The sociodemographic background of this study sample was comparable to that of the National Oral Health Survey. Majority were females (58.8%), were of Malay ethnicity (72.7%), and completed college education (49.7%). There was no information on distribution of patients' location whether they were from urban or rural areas but possibly majority would be from the urban as specialist clinics were located in the major cities of each state. Patients from the sampled clinics were more of the older age group as only one-quarter (25.0%) were aged 34 years and younger; this is consistent with about one-fifth (41.5%) in this same age group of the National Oral Health Survey sample, while the rest were older.

### 2.6. Data Analysis

We tabulated the data and made calculations using Microsoft Excel 2010 (Microsoft, Redmond, WA, USA). We performed statistical analysis using IBM SPSS Statistics software version 20.

## 3. Results

### 3.1. Cost of Periodontitis Management

**Figure 2:**
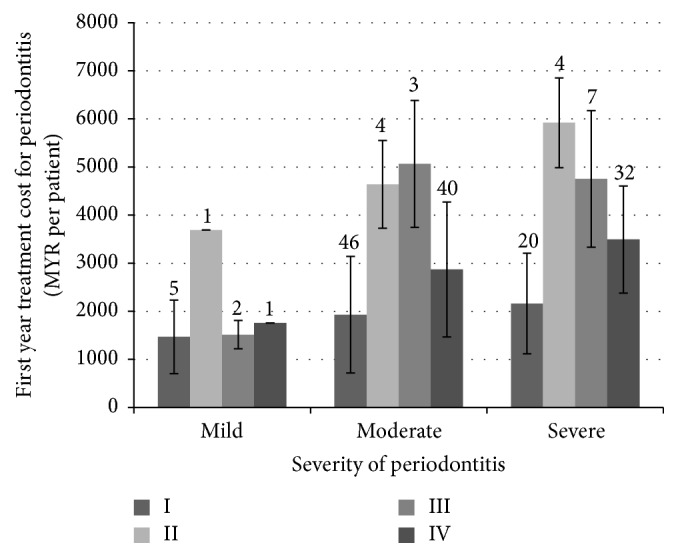
Average treatment cost for periodontitis by treatment mix and periodontitis severity (Total patients = 165). I: nonsurgical only (NSPT), II: NSPT and periodontal surgery (PS), III: NSPT and nonsurgical rehabilitative therapy (NSRT), IV: NSPT, PS, and NSRT.

It costs MYR2820 to manage periodontitis per patient per year and MYR376 per outpatient visit (Table 1). Provider cost contributed 90% to the total cost (Table 1), and cost increased with disease severity (ANOVA; *P* = 0.022) (Figure 2). It costs higher to treat patients who required surgical compared to those requiring nonsurgical treatment alone. (ANOVA; *P* < 0.001) (Figure 2).

### 3.2. Clinical Burden of Periodontitis Based on National Epidemiological Findings and Census Report

Data from the 2010 National Oral Health Survey for Adults estimated the prevalence of moderate and severe periodontitis to be 30.3% and 18.2%, respectively (Table 2); these observations were based on a household survey involving a total of 8,332 dentate adults aged 15 and above nationwide. Using national census data of the Malaysian population aged 15 years and above, the clinical burden for moderate periodontitis was projected to be 1.8 times higher in magnitude compared to severe periodontitis. Cumulatively, almost half of the adult population totalling 11.5 million people suffer from either moderate or severe periodontitis. Prevalence of individuals with mild periodontitis was not captured by the adult survey and, hence, no projection may be made for its population prevalence.

### 3.3. Economic Burden of Periodontitis

The economic burden of managing all cases of periodontitis at the national level from the societal perspective was approximately MYR 32.5 billion, accounting for 3.83% of the present Gross Domestic Product (Table 3). The bulk of the burden may be attributable to moderate periodontitis with a quantum of MYR 18.3 billion. Managing all severe periodontitis patients would cost the nation about MYR 13.7 billion. From the providers' perspectives, the economic burden of managing all cases of periodontitis at the national level from the providers' perspective was approximately MYR 29.1 billion, exceeding year 2011 Ministry of Health budget by 60.6%.

**Figure 3:**
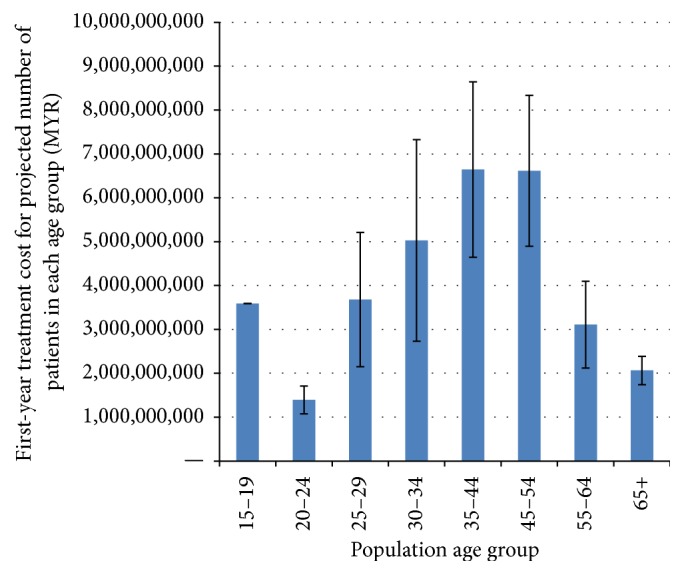
Economic burden of periodontitis by population age group.

**Figure 4:**
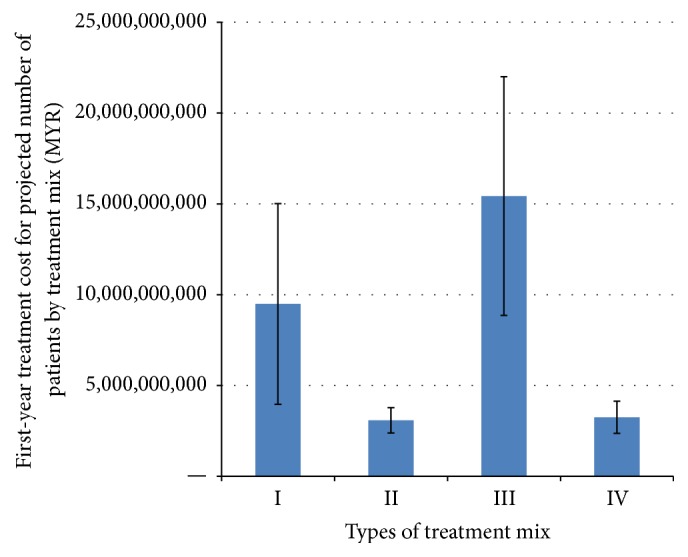
Economic burden of periodontitis by treatment mix. I: nonsurgical only (NSPT), II: NSPT and periodontal surgery (PS), III: NSPT and nonsurgical rehabilitative therapy (NSRT), IV: NSPT, PS, and NSRT.

We also looked at the distributions of economic burden according to age group and type of treatment received by patients. To approximate the national economic burden of periodontitis by age group, we used cost data from the present study and projected the disease prevalence per age group based on the National Oral Health Survey (NOHSA) and national census. The highest burden of care was observed for the 35–44- and 45–54-year age group (approximately MYR 6.6 billion per age group), while the lowest was for the 20–24-year age group (approximately MYR 1.4 billion) (Figure 2). With regard to type of treatment received, the magnitude of economic burden was observed to be highest for patients who received a combination of nonsurgical periodontal therapy and nonsurgical rehabilitative therapy which will cost the government MYR 15.4 billion to treat (Figures 3 and 4). Patients requiring periodontal surgeries, however, demonstrated lowest economic burden for the government.

## 4. Discussion

In spite of the considerable burden that periodontitis poses on individuals, societies, and health care systems, there is still inadequate documentation on the magnitude of its impacts on health expenditure and national economic burden. In publicly funded health care systems, such as the oral health care delivery system in Malaysia, scarce resources limit the feasibility of meeting all patients' needs and wants. Following this, many programmes or treatment modalities may not be funded adequately if deemed to be “less beneficial” to societies and health care funders in comparison to those that yield greater outcomes in terms of maintaining health and prolonging lives. In providing care for periodontitis, it is not known what its magnitude of impacts on a country's economy would be if it is not prevented or left untreated until it has reached an advanced stage.

Periodontitis is a disease which seems unassuming in nature when it is at an early stage. Because of this, most patients will not appreciate the need to seek early treatment. Measuring the economic burden imposed by periodontitis on society as a whole means quantifying the consumption of health care resources and production losses incurred by the disease. In this study, the total estimated cost of managing periodontitis was combined with its epidemiological burden data (prevalence) to calculate its national economic burden. Sources of data were a combination of primary and secondary sources; cost data were collected by authors and reported in another paper [18], while prevalence estimates and national census data were from reliable government documents.

This is the first patient-based study that quantitatively validates the longstanding hypothesis that a heavy economic burden is imposed on health care systems and the society to provide care for patients with periodontitis. We reviewed data from 2010 National Oral Health Survey for Adults and observed the prevalence of moderate and severe periodontitis observed to be 30.3% and 18.2%, respectively. Using national census data of the Malaysian population aged 15 and above, the clinical burden for moderate and severe periodontitis was projected to affect 7.2 and 4.3 million adults, respectively. The cost for managing a patient with periodontitis is substantial at MYR 2820 per patient for one year of treatment and MYR 376 per visit. This cost is comparable to that of managing patients with hypertension [20], stroke [21], and diabetes [22, 23] as studied in various settings in Malaysia.

Consequently, the economic burden of managing all cases of periodontitis at the national level from the providers' perspective was approximately MYR 27.1 billion, exceeding the present Ministry of Health budget by 60.6%. The bulk of the burden may be attributable to moderate periodontitis with a quantum of MYR 16.4 billion; this is explained by the higher prevalence of moderate periodontitis estimated for the adult population. Managing severe periodontitis patients would cost the government about MYR 12.2 billion, which is 72.6% of the present Ministry of Health budget. From the societal perspective, the projected economic burden of managing all cases of periodontitis was even higher at approximately MYR 32.5 billion, accounting for 3.83% of the present Gross Domestic Product. Similarly the bulk of the burden may be attributable to moderate periodontitis with a quantum of MYR 18.3 billion. To manage all severe periodontitis patients would cost the nation about MYR 13.7 billion.

These findings may be used to raise awareness among policy-makers and the public about the negative economic impact of periodontitis and further emphasise the need for oral disease prevention and oral health promotion. For most cases of periodontal disease, causes are known and are preventable. Primary prevention should be emphasised and early detection of signs and symptoms reinforced and targeted to the younger age group. This is crucial because with such an economic burden higher than the health budget itself, and so many health needs of the population to cater for, no country will be able to meet these periodontal treatment needs hence many patients will be left undertreated or untreated. As it is, utilisation of public sector dental clinics including specialist periodontal clinics among adults is already very low. Opting for private dental care is not viable for most of the population given that cost of first-year periodontal therapy (MYR 2,810) is twice the average spending on health per person per year (MYR 1,296). It is worthwhile to note here that up to a quarter of chronic periodontitis patients have some form of chronic diseases such as diabetes and hypertension and hence are already burdened with health care costs of those comorbidities.

The highest burden of care was observed for the 35–44- and 45–54-year age group (approximately MYR 6.6 billion per age group), while the lowest was for the 20–24-year age group (approximately MYR 1.4 billion). While the risk of periodontal disease increases as one ages, periodontitis can have early onset during young adulthood. However, majority of patients would seek treatment when the symptoms get worse and this would be at about 35–44 years old. The magnitude of economic burden was observed to be highest for patients who received a combination of nonsurgical periodontal therapy and nonsurgical rehabilitative therapy which will cost the government MYR 15.4 billion to treat. Patients requiring periodontal surgeries, however, demonstrated lowest economic burden for the government. This could be due to the lower clinical burden associated with patients requiring periodontal surgeries.

This study had focused only on periodontitis management at the specialist periodontal clinics within the purview of the Ministry of Health for the first year of treatment. While it is useful for identifying cost and effectiveness of the specialist programme, its findings are not to be generalised for periodontal care at the primary dental clinics, nor does it include activities related to promotion of oral and periodontal health in school dental service settings and other public setups. This study also did not include management of periodontitis at the private sector as well as the public sector other than that of the Ministry of Health as this was beyond its scope. Hence, interpretation of the economic burden of managing periodontitis must be done in the right context; conversely it could actually be higher than what is estimated in this study.

## 5. Conclusion

This was the first economic study on periodontology conducted in Malaysia as well as the Asia-Pacific region. The economic burden of periodontitis in Malaysia is found to be substantial and comparable with that of other chronic diseases in the country. As illustrated in this study, this high economic burden may mainly be attributed to the high prevalence of the disease and resulting need for specialist periodontal care. The low utilisation of public sector dental service and in the specialist periodontal clinics in particular suggests that the burden of untreated periodontitis is a public health problem and needs to be addressed. In addition, periodontitis patients face a higher risk to also suffer from chronic conditions such as diabetes mellitus and hypertension; this link emphasises the dire need for early detection of periodontitis as well as these systemic conditions so that appropriate and timely care may be obtained.

## Figures and Tables

**Table 1:** Average treatment cost for periodontitis.

	Total cost by components (MYR)		
	Provider cost	Patient cost	Total cost
Per patient/year	2,524	296	2,820
Per outpatient visit	336	39	376

**Table 2:** Clinical burden of periodontitis based on national epidemiological findings and census report.

	Moderate periodontitis	Severe periodontitis	All cases
% of population with periodontitis (MOH, 2012)	30.30%	18.20%	48.5%
Number of adults at risk of periodontitis (aged 15 and above) (National Census Report, 2011)	—	—	23,757,994
Number of adults estimated as having periodontitis	7,198,672	4,323,955	**11,522,627**

**Table 3:** Economic burden by periodontitis severity.

	Moderate periodontitis	Severe periodontitis	All cases
Estimated number with periodontitis	7,198,672	4,323,955	11,522,627
Cost per patient	MYR 2,545	MYR 3,174	MYR 2,820
Economic burden	MYR 18.3 billion	MYR 13.7 billion	MYR 32.5 billion
% of GDP^*∗*^	2.16%	1.62%	3.83%
Provider cost per patient	MYR 2,275	MYR 2,831	MYR 2,352
Economic burden from providers' perspective	MYR 16.4 billion	MYR 12.2 billion	MYR 27.1 billion
% of Ministry of Health budget^*∗∗*^	97.07%	72.56%	160.64%

^*∗*^Malaysia's GDP, 2011 = MYR 847,319,000,000.

^*∗∗*^Ministry of Health budget, 2011 = MYR 16, 870, 767, 600.
